# Effect of sodium bicarbonate contribution on energy metabolism during exercise: a systematic review and meta-analysis

**DOI:** 10.1186/s12970-021-00410-y

**Published:** 2021-02-05

**Authors:** Jorge Lorenzo Calvo, Huanteng Xu, Daniel Mon-López, Helios Pareja-Galeano, Sergio Lorenzo Jiménez

**Affiliations:** 1 Faculty of Physical Activity and Sport science, Universidad Politécnica de Madrid, Madrid, Spain; 2 Faculty of Sport Sciences, Universidad Europea de Madrid, Madrid, Spain; 3 Centre for Sport Studies, Rey Juan Carlos University, Fuenlabrada, Spain

**Keywords:** Sodium bicarbonate, Energy metabolism, exercise, Aerobic-based, Anaerobic-based

## Abstract

**Background:**

The effects of sodium bicarbonate (NaHCO_3_) on anaerobic and aerobic capacity are commonly acknowledged as unclear due to the contrasting evidence thus, the present study analyzes the contribution of NaHCO_3_ to energy metabolism during exercise.

**Methods:**

Following a search through five databases, 17 studies were found to meet the inclusion criteria. Meta-analyses of standardized mean differences (SMDs) were performed using a random-effects model to determine the effects of NaHCO_3_ supplementation on energy metabolism. Subgroup meta-analyses were conducted for the anaerobic-based exercise (assessed by changes in pH, bicarbonate ion [HCO_3_^−^], base excess [BE] and blood lactate [BLa]) vs. aerobic-based exercise (assessed by changes in oxygen uptake [VO_2_], carbon dioxide production [VCO_2_], partial pressure of oxygen [PO_2_] and partial pressure of carbon dioxide [PCO_2_]).

**Results:**

The meta-analysis indicated that NaHCO_3_ ingestion improves pH (SMD = 1.38, 95% CI: 0.97 to 1.79, *P* < 0.001; I^2^ = 69%), HCO_3_^−^ (SMD = 1.63, 95% CI: 1.10 to 2.17, *P* < 0.001; I^2^ = 80%), BE (SMD = 1.67, 95% CI: 1.16 to 2.19, *P* < 0.001, I^2^ = 77%), BLa (SMD = 0.72, 95% CI: 0.34 to 1.11, *P* < 0.001, I^2^ = 68%) and PCO_2_ (SMD = 0.51, 95% CI: 0.13 to 0.90, *P* = 0.009, I^2^ = 0%) but there were no differences between VO_2_, VCO_2_ and PO_2_ compared with the placebo condition.

**Conclusions:**

This meta-analysis has found that the anaerobic metabolism system (AnMS), especially the glycolytic but not the oxidative system during exercise is affected by ingestion of NaHCO_3_. The ideal way is to ingest it is in a gelatin capsule in the acute mode and to use a dose of 0.3 g•kg^− 1^ body mass of NaHCO_3_ 90 min before the exercise in which energy is supplied by the glycolytic system.

## Background

Energy supply is an important prerequisite for maintaining exercise, in which fat, carbohydrate (glucose) and protein are converted into adenosine triphosphate (ATP) to provide energy for the body. Energy output from human movement is divided between anaerobic and aerobic energy supply systems. The anaerobic systems are the phosphagen system and glycolytic system, which synthesize ATP without oxygen participation. The energy supply substrates of the phosphagen system are ATP and creatine phosphate (CP or phosphocreatine [PCr]), also called the ATP-CP system. ATP-CP participates in energy supply directly, which is the fastest but also shortest way to maintain the duration of the energy supply. The energy substrate of the glycolytic system is glucose, which synthesizes ATP by decomposing glucose. The process by which the body decomposes a substrate under aerobic conditions is called intracellular respiration. This process requires the participation of oxygen, and is called the oxidative system. The mitochondria in the cells are the organs that produce ATP by glucose, fat and protein oxidation, and at the same time, the cardiovascular and respiratory systems need to transport large amounts of oxygen to the muscles for their needs [1], (Table 1).

**Table 1:** The basic characteristics of the three energy supply systems

Name of energy supply system	Energy substrate	Available exercise time	Supply substances and metabolites for ATP recovery
ATP-CP	ATP	6 ~ 8 s	CP
	CP	< 10s	CP + ADP → ATP + C
Glycolytic system	Glucose	2 ~ 3 min	Glucose → Lactic acid
Oxidative system	Glucose	3 ~ 5 min	Glucose → CO_2_ + H_2_O
	Fat	1 ~ 2 h	Fat → CO_2_ + H_2_O

In exercise physiology the interconnection between the energy required to complete different types of exercise and the ways supplied by each energy system together is referred to as the Continuous unity of energy (CUE) [2]. It describes the corresponding overall relationship between different movements and different energy supply paths of the energy system (Unity of sport and energy supply). The CUE is generally expressed as the percentage of aerobic and anaerobic energy supplied. According to the ratio of anaerobic and aerobic energy supplied for different sports, the relative positions of various sports in the CUE can be determined and the sport can be understood by what is the leading energy supply system.

The ratio of the anaerobic and aerobic energy supply is determined by exercise intensity. The ATP-CP system mainly provides energy for high-intensity short-term exercise (i.e., sprinting, throwing, jumping and weight lifting); the glycolytic system mainly provides energy for medium-high-intensity, short-term exercise (i.e., 400 m running and 100 m swimming) and the oxidative system mainly functions for low-medium-intensity, medium-long time exercise (i.e., long distance running, rowing and cycling), (Table 2). The energy supply capacity of different energy systems determines the strength of exercise capacity.

**Table 2:** Corresponding position of sports in the CUE

Sports	Aerobic (%)	Anaerobic (%)	Sports
Weight lifting, diving, gymnastics	0	100	100 m running, Golf and tennis swing
200 m running, wrestling, Ice hockey	10	90	Soccer, basketball, baseball,
	20	80	Volleyball, 500 m skiing, 400 m running
Tennis, lawn hockey	30	70	Lacrosse
800 m running, boxing	40	60	200 m swimming, 1500 m skating
	50	50	
2000 m rowing	60	40	1500 m running
1500 m running, 400 m swimming	70	30	800 m swimming
3000 m running	80	20	Trail running
5000 m running, 10,000 m skating, 10,000 m running, marathon	90	10	Cross country skiing, jogging
	100	0	

The ATP-CP system tells us that when ATP is used, creatine kinase decomposes PCr and simultaneously removes inorganic phosphate (Pi) to release energy during explosive activities [3]. The energy generated when decomposing PCr can combine Pi with adenosine diphosphate (ADP) to regenerate ATP, thereby maintaining the stability of ATP levels. The principle of the glycolytic system is that glycogen or glucose decomposes to form pyruvate, which becomes lactic acid in the absence of oxygen. If lactic acid is not removed in time, it will be decomposed and converted into lactate and cause a large amount of H^+^ accumulation, resulting in muscle acidification, causing acidosis [4].

The increase of H^+^ will cause decreases of pH in the body, and the destroyed acid-base balance will damage muscle contractility and hinder ATP production. In order to reduce the effect of free H^+^, alkaline substances in blood and muscle will combine with H^+^ to buffer or neutralize it [5]. In the body, there are three main chemical buffers, bicarbonate ions (HCO_3_^−^), Pi, and protein. In addition, the hemoglobin in red blood cells is also an important buffer, but a large part depends on HCO_3_^−^ (see Table 3) [1]. When lactic acid is formed, the body’s fluid buffer system will increase the HCO_3_^−^ in the blood to help the body quickly recover from fatigue. This process is called bicarbonate loading [6]. Sodium bicarbonate (NaHCO_3_) is a type of physiological supplement. Ingesting some substances that can increase the HCO_3_^−^ in the blood, like NaHCO_3_, can increase the blood pH and make it more alkaline. The higher the HCO_3_^−^, the stronger the acid-base buffer provided, allowing higher concentrations of lactic acid in the blood.

**Table 3:** Buffer capacity of blood components

Buffers	Slykes*	%
HCO_3_^−^	18.0	64
Hemoglobin	8.0	29
Protein	1.7	6
Pi	0.3	1
Total	28.0	100

Note: * refers to the pH value per liter of blood ranging from 7.4 to 7.0, which can neutralize the milliequivalent of H^+^

There are some studies showing that NaHCO_3_ can change the content of blood lactate (BLa), HCO_3_^−^, pH and BE [7–10] during anaerobic-based exercise. Although those parameters are affected by ingesting NaHCO_3_, the change of anaerobic metabolism systems (AnMS) is different. The capacity of the glycolytic system could increase [11] or stay the same [12], but the ATP-CP system seems not affected by ingestion of NaHCO_3_, because the ATP or PCr content is not affected by NaHCO_3_ [12, 13]. Due to the participation of oxygen in the process of ATP synthesis in the oxidative system, a large number of studies have shown that enhancing oxygen uptake and the muscle’s ability to use oxygen can improve the oxidative system capacity. For that reason, some researchers explored whether NaHCO_3_ will increase oxygen uptake and affect the oxidative system. Similar to the glycolytic system, contradictory evidence is shown in the existing literature, demonstrating that the capacity of the glycolytic system could increase [14] or stay the same with NaHCO_3_ ingestion [15].

The main reason why NaHCO_3_ has different effects on different energy metabolism systems may be due to the different exercise durations reflected by different exercise types. Some studies have shown that an intake of NaHCO_3_ will improve high-intensity intermittent exercise [16, 17] or repeat sprint ability [18, 19]. According to the exercise duration reflected by the specific sport characteristics, some scholars have concluded that NaHCO_3_ has an effect on exercises of less than 4 min, but no effect on exercise of a longer duration [20]. Other scholars have found a more specific time effect, that for less than 1 min or more than 7 min it is ineffective and its supplementary benefits for anaerobic exercise within 2 min are very limited [1]. Another point is the gender difference, that men seem to benefit more from the supplementation of NaHCO_3_ [19, 21], the reason for which might be found in physiological differences. Women have smaller type II fibers than men, and type II fibers rely predominantly on the glycolytic energy system [22]. This may explain why the previous research has contradictory results.

Unlike previous studies, due to different results and study discussions, this review no longer focuses on specific sports, exercise tasks or duration, but instead goes back to its source to explore the mechanism and principles of application of NaHCO_3_. Despite all apparent changes, the energy supply is essentially the same in all sports.

Knowledge of nutrition can influence dietary choices and impact athletic performance, and is important for coaches because they are often the most significant source of such knowledge for their athletes [23]. In addition, one article concluded that the level of athletes’ knowledge about the proper and intended use of sports supplements reveals the necessity of enforcing ongoing education about sports supplementation [24]. Clarifying the role of NaHCO_3_ can provide a reference for a lot of athletes and coaches.

## Materials and methods

### Search strategy

The present article is a meta-analysis focusing on the contribution of sodium bicarbonate to energy metabolism during different types of exercise (i.e., aerobic-based and anaerobic-based). This study followed the Preferred Reporting Elements for Systematic Reviews and Meta-analysis (PRISMA) guidelines [25] and the eligibility criteria of articles was determined with the application of the Participants, Intervention, Comparison, Outcome and Study design (PICOS) question model [26], elements were used in title, abstract and/or full text of articles to identity studies that met the eligibility criteria (Table 4).

**Table 4:** PICOS (Participants, Intervention, Comparison, Outcomes and Study design)

PICOS components	Detail
Participants	Healthy exercise adults
Intervention	Supplementation with NaHCO_3_
Comparison	Same conditions with placebo or control group
Outcomes	Changes in some parameters that can express changes in energy metabolism (i.e., HCO_3_^−^, pH, BE, BLa, VO_2_, CO_2_, PO_2_ and PCO_2_)
Study design	Crossover or counterbalanced double- or single-blind, randomized controlled trials

A systematic search was conducted using PubMed, Web of Science, SCOPUS, Medline and SPORTDiscus databases to identify eligible studies published from 2010 to June 2020. Search terms related with main concepts were used: “sodium bicarbonate” AND (“metabolism” OR “energy expenditure”) AND (“exercise” OR “physical activity” OR “sport”) AND “aerobic” AND “anaerobic”. Through this search, a total of 351 articles were obtained and 17 articles were finally included in this meta-analysis.

### Selection of articles: inclusion and exclusion criteria

**Figure 1:**
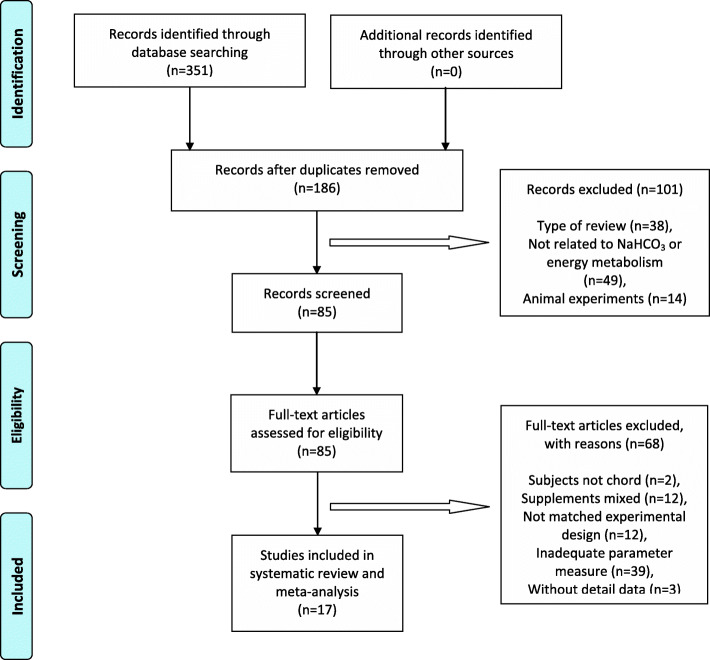
Fig. 1 PRIMA flow chart of selection process for articles included in this meta-analysis

After obtaining the 351 articles according to the inclusion criteria of PICOS in the search, the following exclusion criteria were taken into consideration to determine the final studies: 1) Review and meta-analysis; 2) No sodium bicarbonate supplement ingestion or the outcomes measure not related to energy metabolism; 3) Supplement mixed with other supplements (i.e. caffeine or beta-alanine); 4) Animal experiments; 5) Injury participants or without training experience; 6) Study design not matched: not under the same experimental conditions (i.e., Hypoxia or ingested after high intensity exercise), without exercise after ingesting, no placebo as a comparison group; 7) Inadequate parameter measurement; 8) Data not described in detail (e.g., no mean or standard deviation (SD), no response after emailing author). The data collection process is presented in Fig. 1.

The methodological quality of the articles, was evaluated using McMaster’s Critical Review Form [27]. The McMaster Form contains 15 items that are scored depending on the degree to which the specific criteria were met (yes = 1, no = 0). A summary score was calculated for each article by summing the total score obtained across relevant items and dividing it by the total possible score. The evaluation score of the quality of the articles is shown in Table 5. The main deficiencies found in methodological quality are associated with item 14 of the questionnaire, which is “were drop-outs reported?”, as there is no description about whether participants dropped out or not.

**Table 5:** Methodological quality of the studies included in this meta-analysis [27]

	Items	1	2	3	4	5	6	7	8	9	10	11	12	13	14	15	T(s)	%	MQ
**References**	[33]	1	1	1	1	1	1	1	1	1	1	1	1	1	0	1	14	93.3	VG
	[29]	1	1	1	1	1	1	1	1	1	1	1	1	1	1	1	15	100	E
	[39]	1	1	1	1	1	1	1	1	1	1	1	1	1	1	1	1	100	E
	[28]	1	1	1	1	1	1	1	1	1	1	1	1	1	0	1	14	93.3	VG
	[40]	1	1	1	1	1	1	1	1	1	1	1	1	1	1	1	15	100	E
	[41]	1	1	1	1	1	1	1	1	1	1	1	1	1	0	1	14	93.3	VG
	[42]	1	1	1	1	1	1	1	1	1	1	1	1	1	0	1	14	93.3	VG
	[43]	1	1	1	1	1	1	1	1	0	1	1	1	1	0	1	13	86.7	VG
	[32]	1	1	1	1	1	1	1	1	1	1	1	1	1	0	1	14	93.3	VG
	[31]	1	1	1	1	1	1	1	1	1	1	1	1	1	0	1	14	93.3	VG
	[44]	1	1	1	1	1	1	1	1	1	1	1	1	1	0	1	14	93.3	VG
	[45]	1	1	1	1	1	1	1	1	1	1	1	1	1	0	1	14	93.3	VG
	[46]	1	1	1	1	1	1	1	1	1	1	1	1	1	1	1	15	100	E
	[30]	1	1	1	1	1	1	1	1	1	1	1	1	1	1	1	15	100	E
	[47]	1	1	1	1	1	1	1	1	1	1	1	1	1	1	1	15	100	E
	[48]	1	1	1	1	1	1	1	1	1	1	1	1	1	0	1	14	93.3	VG
	[49]	1	1	1	1	1	1	1	1	1	1	1	1	1	1	1	14	93.3	VG
**T(i)**		17	17	17	17	17	17	17	17	16	17	17	17	17	6	17		M = 95.28	

T(s): Total items fulfilled by study. (1) Criterion met; (0) Criterion not met. T(i): Total items fulfilled by items. Methodological Quality (MQ): poor (P) ≤ 8 points; acceptable (A) 9–10 points; good (G) 11–12 points; very good (VG) 13–14 points; excellent (E) =15 points. M refers to mean

### Data extraction and analysis

Physiological results data were extracted in the form of mean, SD, and sample size for placebo and NaHCO_3_ cohorts. Data were collected directly from tables or within text of the selected studies when possible. Data of 6 studies were partially abstracted by online graph digitizing software (WebPloDigitizer^1^) when values were not reported in the text. This included values abstracted directly with mean and SD [28–30] or calculated after obtaining mean and standard error (SE) [31, 32] or a 95% confidence interval (95% CI) [33]. A study was excluded from the meta-analysis when the missing data could not be provided, or the author did not respond [34–36]. Dependent variables include those parameters relevant to energy metabolism after exercises following the supplement intervention. When pertinent data were not available or referenced in the article, the study was excluded from the meta-analysis.

The meta-analysis was conducted using the Review Manager 5.3 (v5.3, Cochrane Collaboration, Copenhagen, Denmark, 2020) in order to aggregate, via a random-effects model [37], the standardized mean difference (SMD) between the effects of NaHCO_3_ and placebo cohorts. The mean ± SD and sample size were used to calculate SMD. A sub-group analysis was also performed to evaluate the influence on exercise with different metabolic characteristics. The use of the SMD summary statistic allowed all effect sizes to be transformed into a uniform scale, which was interpreted, according to Cohen’s conventional criteria [38], with SMD of < 0.20 being classified as negligible, 0.20–0.49 classified as small; 0.50–0.79 classified as moderate; and > 0.80 classified as large. Heterogeneity was determined using I^2^ value, with values of 25, 50 and 75 indicating low, moderate and high heterogeneity, respectively. The results are reported as weighted means and 95% CI. The statistical significance was set at *p* < 0.05.

## Results

### Study selection and characteristics

A total of 351 articles were initially identified through databases. Of the 186 that remained after the removal of 165 duplicates, 101 articles were not considered relevant and were excluded. Based on the inclusion criteria, 17 articles, published between 2010 and 2019, met the full set of criteria and were included for review. All descriptions and characteristics of the review studies are presented in Tables 6 and 7. Moreover, the quality assessment of selected articles was classified as Very Good (Table 5).

**Table 6:** General characteristics of the studies included (Exercise characteristics as anaerobic-based)

References	Study Design	Population characteristics	Intervention	Supplement situation	Experimental design	Physiological Results	Performance results
[33]	Randomized double-blind crossover	12 M: elite BMX cyclists, age: 19.2 ± 3.4 y, height: 174.2 ± 5.3 cm and BM: 72.4 ± 8.4 kg	0.3 g•kg^− 1^ BM of NaHCO_3_ or 0.045 g•kg^−1^ BM of NaCI (PLA)	Ingested 90 min before the trial in gelatin capsules once	3 races of BMX (track length of 400 m) with 15 min interval	↑HCO_3_^−^, ↑pH and ↑BE vs. PLA, (12.95 ± 1.3, 7.2 ± 0.05 and − 12.66 ± 3.13 vs. 11.45 ± 1.3, 7.14 ± 0.05 and − 16.27 ± 3.18), =BLa, =HR, =RPE, =VO_2_, =VCO_2_ and = VE vs. PLA	=Time, = Velocity peak (VP) and = Time to VP vs. PLA
[29]	Double-blind counterbalanced crossover	18 M: rugby, judo (*n* = 2) and jiu-jitsu (*n* = 5), age: 26 ± 5 y; BM: 88.8 ± 6.8 kg; height: 1.78 ± 0.07 m;	500 mg/kg BM of CL or NaHCO_3_ or CACO_3_ (PLA) Divided into four individual doses of 125 mg/kg BM	Last one within 4 h before trial, ingested in gelatin capsules for 5 consecutive days	4 bouts of the upper body WAnT with 3 min interval	=HCO_3_^−^, =pH, =BE, =BLa vs. other conditions	↑ TWM (2.9%) and ↑ 3rd + 4th of Wingate (5.9%) vs. CL and PLA =1st + 2nd of Wingate
[39]	Randomized crossover	10 M: age: 22 ± 4 y, height: 1.77 ± 0.06 m, BM: 76 ± 9 kg.	0.5 g•kg^− 1^ BM of NaHCO_3_ or 0.2 g•kg^− 1^ BM of NaCI (PLA) Divided into 3 doses	Each dose at 4 h interval on experimental day, ingested as NR once	2 WAnT with 5 min interval	↑HCO_3_^−^,↑pH and ↑BE vs. PLA (12.7 ± 1.3, 7.22 ± 0.04 and − 13.7 ± 1.8 vs. 9.5 ± 1.7, 7.15 ± 0.05 and − 17.8 ± 2.1), =BLa vs PLA	↑ Work completed (5 ± 4%) vs. PLA, = Rate of fatigue vs. PLA =PP (↓PLA, −8 ± 8%)
[28]	Randomized double-blind crossover	13 M: elite swimmers, age: 20.5 ± 1.4 y, BM: 80.1 ± 8.1 kg, height: 188 ± 8 cm	0.3 g•kg^− 1^ BM of NaHCO_3_ or CACO_3_ (PLA)	Ingested 60 min before the trial in gelatin capsules once	Two 100 m freestyle sprints with 12 min interval	↑HCO_3_^−^, ↑pH and ↑BE vs. PLA (10.61 ± 3.43, 7.15 ± 0.05 and − 18.68 ± 2.91 vs. 7.77 ± 2.41, 7.05 ± 0.06 and − 22.78 ± 2.21), =BLa vs. PLA	= 1st 100 m swim vs. PLA, ↓ Time of 2nd 100 m swim vs. PLA (1.5 s)
[40] a	Randomized, double-blind, counterbalanced	12 M: resistance-trained participants (age: 20.3 ± 2 y, BM:88.3 ± 13.2 kg, height:1.80 ± 0.07 m)	0.3 g•kg^− 1^ BM of NaHCO_3_ or CACO_3_ (PLA) Divided into 4 equal doses	Each dose consumed at 10 min intervals, 1st dose at 80 min before the trial, ingested in gelatin capsules once	4 sets of SQ, LP and KE with 120 s interval, 10-12RM per set with 90s interval	↑HCO_3_^−^, ↑pH, ↑BE and ↑BLa vs. PLA (17.86 ± 3.63, 7.35 ± 0.04, − 7.67 ± 4.16 and 17.16 ± 2.09 vs. 14.19 ± 2.62, 7.09 ± 0.03, − 11.5 ± 3.2 and 12.49 ± 2.45)	↑ Total volume (SQ + LP + KE + PT) vs. PLA (163.7 ± 15.1 vs. 156.7 ± 14.5)
[41]	Double-blind, counterbalanced	21 M, age: 25 ± 5 y, BM: 80.7 ± 10.6 kg, height: 1.79 ± 0.06 m	0.3 g•kg^− 1^ of BM NaHCO_3_ or maltodextrin (PLA)	Ingested 0.2 g•kg^− 1^ BM alongside the breakfast, 0.1 g•kg^− 1^ BM 2 h before the trial in gelatin capsules once	A habituation trial of the cycle-capacity test to exhaustion at 110% of Wmax	↑HCO_3_^−^, ↑pH, ↑BE and ↑BLa vs. PLA (15.26 ± 2.78, 7.28 ± 0.05, − 9.6 ± 3.38 and 14.5 ± 2.9 vs. 12.82 ± 2.1, 7.23 ± 0.06, − 12.69 ± 2.8 and 12.4 ± 2)	=TWD, except participants who have GI ↑TWD vs. PLA (48.4 ± 9.3 vs. 46.9 ± 9.2)
[42]a	Randomized double-blind counterbalanced crossover	20 rowers: age: 23 ± 4 y, height: 1.85 ± 0.08 m, BM: 82.5 ± 8.9 kg,	0.3 g•kg^− 1^ BM of NaHCO_3_ or maltodextrin (PLA)	Ingested 0.2 g•kg^− 1^ BM 4 h before and 0.1 g•kg^− 1^ BM 2 h before the trial as NR once	2000 m rowing-ergometer TTs	↑HCO_3_^−^, ↑pH, ↑BE and ↑BLa vs. PLA (10.56 ± 1.75, 7.18 ± 0.06, − 15.56 ± 2.69 and 16,5 ± 0.9 vs. 9.1 ± 1.71, 7.12 ± 0.07, − 18.13 ± 2.77 and 14.1 ± 0.9)	= Time of 1st and 2nd 500 m, ↓ Time of 3rd and 4th 500 m (0.5 ± 1.2 s and 1.1 ± 1.7 s)
[43]	Randomized, single-blind, counterbalanced	14 swimmers (6 M, hight:181.2 ± 7.2 cm; BM: 80.3 ± 11.9 kg, 8F, height: 168.8 ± 5.6 cm; BM: 75.3 ± 10.1 kg)	0.3 g•kg^− 1^ BM of NaHCO_3_ or 0.045 g•kg^− 1^ BM of NaCI (PLA)	Ingested 2.5 h before the trial as NR once	Completed 8x25m front crawl maximal effort sprints with 5 s interval	↑HCO_3_^−^, ↑pH, ↑BE and ↑BLa vs. PLA (16 ± 0.05, 7.26 ± 0.01, − 11.1 ± 0.08 and 17.69 ± 1.06 vs. 13.8 ± 0.6, 7.2 ± 0.02, − 14.6 ± 1.1 and 14.62 ± 1.25), ↓K+ vs PLA (3.8 ± 0.1 vs. 4.4 ± 0.1), =NA^+^	↓Total swim time (2%) vs. PLA
[32]	Randomized, double-blind, counterbalance crossover	10 elite BMX riders, age: 20.7 ± 1.4 y, height: 178.3 ± 2.1 cm and BM: 77.9 ± 2.1 kg,	0.3 g•kg^− 1^ BM of NaHCO_3_ or placebo (PLA)	Ingested 90 min before the trial in gelatin capsules once	3x30s Wingate tests with 15 min interval	↑HCO_3_^−^, ↑pH and ↑BE vs. PLA (10.17 ± 1.77, 7.22 ± 0.09 and 16.57 ± 3.51 vs. 6.88 ± 2.78, 7.09 ± 0.03 and − 22.49 ± 1.39), =BLa vs. PLA	=PP, = Time to PP, =Mean power, =Fatigue index vs. PLA
[31]	Randomized double-blind counterbalanced	11 trained cyclists (10 M and 1F), age: 24.5 ± 2.8 y, height: 1.78 ± 2.7 m and BM: 73.2 ± 3.8 kg	0.3 g•kg^− 1^ BM of NaHCO_3_ or 0.2 g•kg^− 1^ BM of CaCO_3_ (PLA)	Ingested 90 min before the trial in gelatin capsules once	70s supramaximal exercise	↑HCO_3_^−^, ↑pH and ↑BE vs. PLA (19.53 ± 3.98, 7.3 ± 0.03 and − 6.15 ± 3.91 vs. 15.12 ± 3.15, 7.21 ± 0.07 and − 12.67 ± 3.81), =BLa, =VO_2_, =VCO_2_, =VE, =PO_2_, ↑PCO_2_ vs. PLA (42 ± 2.99 vs. 38.9 ± 3.65)	↑P50 and ↑Ptot vs. PLA (469.6 ± 28.6 and 564.5 ± 29.5 vs. 448.2 ± 7.7 and 549.5 ± 29.1), =P20 and = Fatigue index vs. PLA
[44]	Randomized double-blind crossover	11 M trained cyclists, age: 32 ± 7.2 y; BM: 77.0 ± 9.2 kg	0.3 g•kg^− 1^ BM of NaHCO_3_ or 0.21 g•kg^− 1^ BM of NaCI (PLA)	Ingested 70-40 min before trial (depending on individual time to peak pH) as NR once	3 min all-out critical power test	=HCO_3_^−^, =H^+^, =BLa, =PO_2_ and = PCO_2_ vs. PLA	↑TWD (5.5%) and ↑W′ (14%) vs. PLA, =CP vs. PLA

“a” means the energy metabolism mixed aerobic and anaerobic in this experimental trial, the ratio of aerobic and anaerobic almost half and half, and the physiological results relevant with anaerobic-based, so classified into anaerobic-based exercise

Notes for Tables 5 and 6: All variables of physiological results are mainly reflected in the change of the end point value (i.e., The influence after the last exercise if it has two or more bouts)

Abbreviations for Tables 5 and 6:

Supplements: NaHCO_3_: sodium bicarbonate, NaCI: sodium chloride, CI: calcium lactate, CaCO_3_: calcium carbonate, NaAc: trihydrate, NH_4_CI: ammonium chloride, PLA: placebo

Physiological abbreviations: M: male, F: female, BM: body mass, BLa: blood lactate, BE: base excess, ABE: actual base excess, HR: heart rate, RPE: rate of perceived exertion, VO_2_: oxygen uptake, VCO_2_: carbon dioxide production, VE: pulmonary ventilation, RER: respiratory exchange rate, FFA: free fatty acid, CHO: carbohydrate, PO_2_: oxygen partial pressure, PCO_2_: carbon dioxide partial pressure

Performance abbreviations: TWM: total mechanical work, PP: peak power, PO: power output, CP: curtail power, SQ: back squats, LP: inclined leg presses, KE: knee extensions, PT: performance test, WAnT: Wingate Anaerobic Test, TWD: total work done, TTE: time to exhaustion, FHST: Field hockey skill test, LIST: Loughborough intermittent shuttle test, P20: power output during 1st 20s, P50: power output during last 50s, Ptot: total power output

Others: NR: not recorded, BMX: bicycle motocross, w’: curvature constant, IAT: individual anaerobic threshold, ↑: Significantly higher, ↓: Significantly lower, =: no significant difference

**Table 7:** General characteristics of the studies included (Exercise characteristics as aerobic-based)

References	Study Design	Population characteristics	Intervention	Supplement situation	Experimental design	Physiological Results	Performance results
[45]	Randomized single-blind crossover	6 M: age 24 ± 4 y, height: 1.81 ± 0.10 m, and BM: 73.92 ± 11.46 kg)	4 mmol•kg^− 1^ BM of NaHCO_3_ or NaAc Divided into two equal doses with 45 min interval	Last dose ingested 90 min before trial as NR once	Cycling for 120 min at 119 ± 16 W (~ 50% VO_2_peak)	↑blood glucose and ↑Fat oxidation vs. NaAc (3.59 ± 0.45 and 0.11 ± 0.03 vs. 3,21 ± 0.43 and 0.07 ± 0.02) =VO_2_, =VCO_2_, =RER, =BLa vs. NaAc	NR
[46]	Randomized double-blind crossover	8 M: well-trained cyclists and triathletes, age: 31.4 ± 8.8 y, height: 184.6 ± 6.5 cm, BM: 74.1 ± 7.4 kg,	0.3 g•kg^− 1^ BM of NaHCO_3_ or 0.045 g•kg^− 1^ BM of NaCI (PLA)	Ingested 90 min before the trial as tablets for 5 consecutive days	Maintain constant-load cycling at ‘CP’ as long as possible	↑HCO_3_^−^, ↑pH and ↑ABE vs. PLA (32.6 ± 2.7, 7.48 ± 0.02 and 8.3 ± 2.3 vs. 26 ± 1.1, 7.43 ± 0.02 and 2.0 ± 09), =Na^+^, =VO_2_, =VCO_2_, =RER and = HR vs. PLA	=CP vs. PLA ↑TTE (23.5%)
[30]	Double-blind counterbalanced	11 trained cyclists, age: 35.7 ± 7.1 y, BM: 74.7 ± 10.0 kg, height: 1.75 ± 0.10 m	0.15 g•kg^− 1^ BM of NH_4_CI or 0.03 g•kg^− 1^ BM of NaHCO_3_ or 0.15 g•kg^− 1^ BM of CaCO_3_ (PLA)	Ingested 100 min before the trial in gelatin capsules once	4-km cycling	↑pH vs. PLA (7.3 ± 0.1 vs. 7.2 ± 0.07), =HCO_3_^−^, =BE, =BLa, =VO_2_, =VCO_2_, =PO_2_, =PCO_2_ and = RPE vs. PLA	=PO, =Anaerobic PO and = Aerobic PO vs. PLA
[47]	Randomized double-blind crossover	21(16 M, 5F) well-trained cyclists: age: 24 ± 8 y, BMI: 21.3 ± 1.7 kg/m^2^	0.3 g·kg^− 1^ BM of NaHCO_3_ or 4 g NaCI (PLA)	Ingested 2 h–1 h before the trial as NR once	30 min cycling at 95% IAT followed by exercising at 110% IAT until exhaustion	↑HCO_3_^−^, ↑pH and ↑BE vs. PLA (27.6 ± 1.7, 7.45 ± 0.03 and 3.1 ± 1.6 vs. 21.4 ± 2, 7.38 ± 0.03 and − 2.6 ± 1.7), =BLa. =HR, =PCO_2_, ↓PO_2_ vs. PLA (83.4 ± 6.5 vs. 88 ± 6.2)	↑TTE vs. PLA (49.5 ± 11.5 min vs. 45.0 ± 9.5 min)
[48]	Randomized single-blind crossover	8F elite hockey players, age: 23 ± 5 y, BM: 62.6 ± 8.4 kg, Height: 1.66 ± 0.05 m	0.3 g•kg^− 1^ BM of NaHCO_3_ or 0.02 g•kg^− 1^ BM of maltodextrin (PLA)	Ingested 2/3 of NaHCO_3_ 180 min before and 1/3 90 min before the trials in gelatin capsules once	FSHT+ 2 LIST+FSHT+ 10 min recovery+ 2 LIST+FHST, about 75 min total	↑HCO_3_^−^, ↑pH and ↑BE vs. PLA (21.7 ± 2.9, 7.41 ± 0.05 and − 2.3 ± 3.1 vs. 16.8 ± 1.6, 7.34 ± 0.06 and − 7.9 ± 1.8), =Bla, =glucose, =HR vs. PLA	= Performance time and = Sprint time vs. PLA
[49]	Randomized double-blind crossover	9 M, college tennis players age 21.8 ± 2.4 y; height 1.73 ± 0.07 m	0.3 g•kg^− 1^ BM of NaHCO_3_ or 0.209 g•kg^− 1^ BM of NaCI (PLA)	Ingested before 90 min of trial as NR for once	Tennis simulated match, about 50 min	↑HCO_3_^−^ and ↑BE vs. PLA (37.98 ± 3.15 and 11.36 ± 3.7 vs. 26.37 ± 3.5 and 0.12 ± 2.15), =pH, =BLa, =hematocrit, =HR and = RPE vs. PLA	= Sport skill performance vs. PLA

The study design, testing parameters and participants’ characteristics for the meta-analyzed studies are displayed in Tables 6 and 7. All studies are divided into two types of exercise, either anaerobic-based or aerobic-based. Exercise characteristics depend on the experimental design after NaHCO_3_ intervention in these studies, which is whether the exercise is dominated by anaerobic or aerobic ability. After review, 11 articles [28, 29, 31–33, 39–44] were found to belong to anaerobic-based exercise for analysis of AnMS, which are the ATP-CP and glycolytic systems), and 6 articles [30, 45–49] were found to belong to aerobic-based exercise for analysis of the oxidative system.

The total number of participants across all studies was 215. Studies either used mixed-sex samples (3 studies) or included only men (10 studies) or only women (1 study) and another 3 studies did not describe the gender of sample subjects. Out of the 17 included studies, 14 used a NaHCO_3_ dose of 0.3 g•kg^− 1^, two studies used the dose of 0.5 g•kg^− 1^, and one study used the dose of 4 mmol•kg^− 1^ (about 0.336 g•kg^− 1^). The timing of ingestion ranged from 60 min up to 4 h pre-exercise. In some studies, the dose of NaHCO_3_ was provided at one timepoint, with other studies splitting up the total dose at multiple timepoints. The duration of NaHCO_3_ administration was either once or on 5 consecutive days. The type of administration was via gelatin capsules or tablets, but some studies did not report this information (Tables 6 and 7).

### The influence after ingesting NaHCO_3_ on AnMS

Metabolic by-products (e.g., lactic acid) are largely accumulated following the AnMS energy generation process. In the process of dissociating the metabolic by-product, the concentration of H^+^ in body fluids will increase and therefore lower the pH value. In order to reduce the effect of free H^+^, the alkaline substances in blood and muscle will combine with H^+^ to buffer or neutralize H^+^.

Fortunately, cells and body fluids have buffers such as HCO_3_^−^, that can reduce the impact of H^+^. Without the buffers, H^+^ would lower the body’s pH value by 1.5, resulting in cell death. When the intracellular pH value is lower than 6.9, it inhibits the activity of important glycolytic enzymes and reduces the rate of glycolytic and ATP production. When the pH value reaches 6.4, H^+^ will stop any further decomposition of glycogen, causing ATP to rapidly decline until the end of the failure. However, due to the body’s buffering capacity, even during the most strenuous exercise, the concentration of H^+^ can be maintained at a very low level. Even when exhausted, the muscle pH value drops slightly from the steady state of pH 7.1, but it will not drop to a pH below 6.6–6.4 [1].

To sum up, ingesting NaHCO_3_ will neutralize H^+^, thus affecting the content of buffer substances (HCO_3_^−^) in the body and pH, thereby affecting the body’s acid-base balance. Since ingestion of NaHCO_3_ leads to a higher efflux of lactate from the working skeletal muscle to the plasma, BLa can reflect metabolic ability to a certain extent. Therefore, the four variables (i.e., HCO_3_^−^, pH, BE and BLa), at the last time point (i.e., the influence after the last exercise if it has two or more bouts, as with the variable used to analyze the oxidative system) were chosen to assess the influence of NaHCO_3_ on AnMS.

#### Overall meta-analysis of AnMS

**Figure 2:**
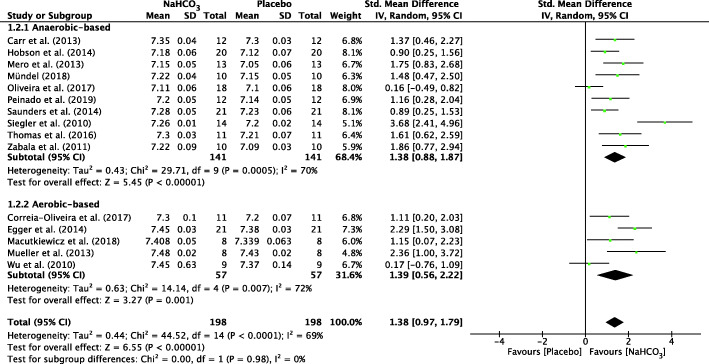
Fig. 2 Forest plot of standardized mean difference (SMD) of NaHCO_3_ vs. placebo on pH after exercise. Squares represent the SMD for each study. The diamonds represent the pooled SMD for all studies. CI: Confidence interval, df: degrees of freedom

**Figure 3:**
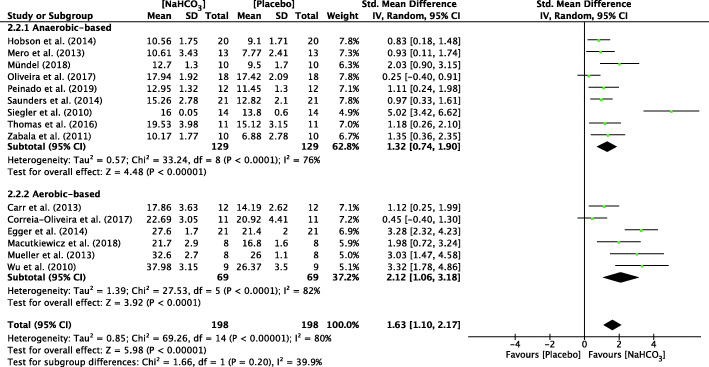
Fig. 3 Forest plot of standardized mean difference (SMD) of NaHCO_3_ vs. placebo on HCO_3_- after exercise. Squares represent the SMD for each study. The diamonds represent the pooled SMD for all studies. CI: Confidence interval, df: degrees of freedom.

**Figure 4:**
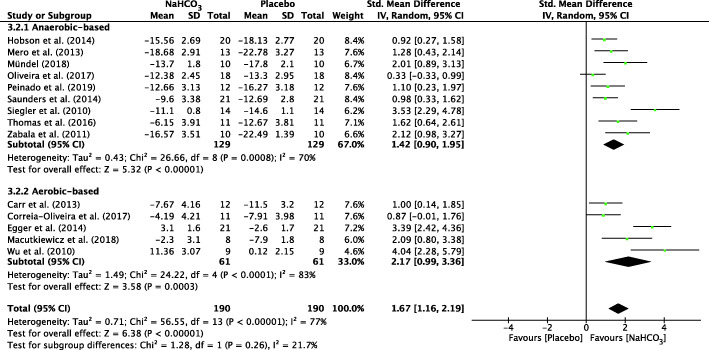
Fig. 4 Forest plot of standardized mean difference (SMD) of NaHCO_3_ vs. placebo on BE after exercise. Squares represent the SMD for each study. The diamonds represent the pooled SMD for all studies. CI: Confidence interval, df: degrees of freedom

**Figure 5:**
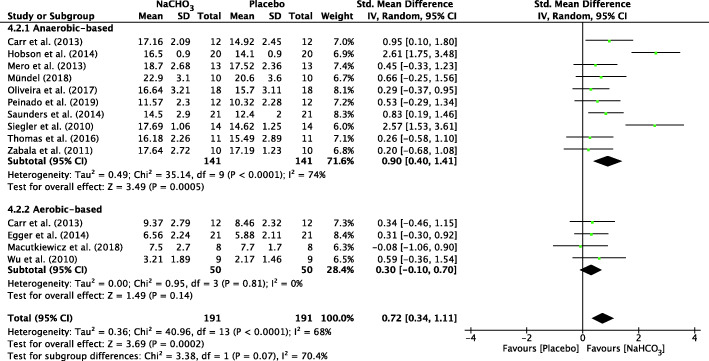
Fig. 5 Forest plot of standardized mean difference (SMD) of NaHCO_3_vs. placebo on BLa after exercise. Squares represent the SMD for each study. The diamonds represent the pooled SMD for all studies. CI: Confidence interval, df: degrees of freedom

The forest plots depicting the individual SMDs and associated 95% CI and random-effect models for pH, HCO_3_^−^, BE and BLa are presented in Figs. 2, 3, 4, 5 respectively.

The SMD for blood pH value was 1.38 (95% CI: 0.97 to 1.79), indicating a significant effect during exercise between NaHCO_3_ and placebo conditions (*p* < 0.001) (Fig. 2). In addition, there was a significant effect during exercise after ingesting NaHCO_3_ on HCO_3_^−^ (SMD = 1.63, 95% CI: 1.10 to 2.17, *P* < 0.001; Fig. 3), BE (SMD = 1.67, 95% CI: 1.16 to 2.19, P < 0.001; Fig. 4) and BLa (SMD = 0.72, 95% CI: 0.34 to 1.11, P < 0.001; Fig. 5) in the blood. Moderate heterogeneity was detected among studies assessing pH (I^2^ = 69%) and BLa (I^2^ = 68%), whereas HCO3- and BE presented a high heterogeneity (I^2^ = 80% and I^2^ = 77% respectively).

#### Sub-group analysis of AnMS

A sub-group analysis was performed to evaluate the effect of NaHCO_3_ ingestion on exercise with different metabolic characteristics. There was a significant difference between two cohorts for pH value in anaerobic-based (SMD = 1.38, 95% CI: 0.88 to 1.87, *P* < 0.001, I^2^ = 70%) and aerobic-based (SMD = 1.39, 95% CI: 0.56 to 2.22, *P* = 0.001, I^2^ = 72%) exercise (Fig. 2). Similar to HCO_3_^−^ and BE, there was a significant difference between two cohorts for HCO_3_^−^ and BE in anaerobic-based exercise (SMD = 1.29, 95% CI: 0.77 to 1.18, *P* < 0.001, I^2^ = 73% and SMD = 1.37, 95% CI: 0.94 to 1.84, *P* < 0.001, I^2^ = 67% respectively) and aerobic-based exercise (SMD = 2.35, 95% CI: 1.06 to 3.64, *P* < 0.001, I^2^ = 83% and SMD = 2.52, 95% CI: 1.07 to 3.96, *P* < 0.001, I^2^ = 84% respectively) (Figs. 3 and 4).

A significant difference between two cohorts was also found for BLa in anaerobic-based exercise (SMD = 0.90, 95% CI: 0.40 to 1.41, *P* < 0.001, I^2^ = 74%) but a non-significant difference on aerobic-based exercise (SMD = 0.30, 95% CI: − 0.1 to 0.7, *P* = 0.14). Heterogeneity was not detected among studies assessing BLa (I^2^ = 0%) in aerobic-based exercise. (Fig. 5).

#### Strategic analysis of NaHCO_3_ in AnMS

**Figure 6:**
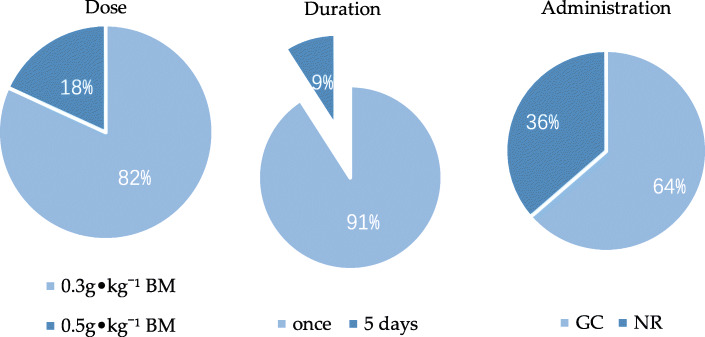
Fig. 6 The percentage of dose, duration and administration of 11 studies based on anaerobic exercise. GC: gelatin capsules, NR: not record

For anaerobic-based exercise (Table 6), 9 (82%) out of 11 studies used 0.3 g•kg^− 1^ BM of NaHCO_3_ and the remaining 2 articles used 0.5 g•kg^− 1^ BM. The duration was once in 10 (91%) studies, while 1 study had duration of 5 consecutive days. The administration of NaHCO_3_ was in gelatin capsules in 7 (64%) studies and not recorded in 4 studies (Fig. 6). More than half of the studies showed NaHCO_3_ ingestion 90–60 min before the trial, other studies shown it more than 2 h before the trial.

### The influence after ingesting NaHCO_3_ on the oxidative system

When performing long-term moderate-intensity exercise, the ventilation volume matches the energy metabolism rate, and it is necessary to constantly change the ratio between the body’s oxygen uptake (VO_2_) and carbon dioxide production (VCO_2_). It is widely acknowledged that a higher VO_2_ is associated with a stronger aerobic capacity. Most of the CO_2_ (about 60–70%) produced during muscle exercise is transported back to the heart in the form of HCO_3_^−^ [1]. CO_2_ and water molecules combine to form carbonic acid, which is unstable and will soon dissolve, forming free H^+^ and HCO_3_^−^:

$$ {\mathrm{CO}}_2+{\mathrm{H}}_2\mathrm{O}\mathbf{\to }{\mathrm{H}}_2{\mathrm{CO}}_3\mathbf{\to}{\mathrm{H}}^{+}+{{\mathrm{H}\mathrm{CO}}_3}^{-}, $$

When the blood enters the area where the partial pressure of carbon dioxide (PCO_2_) in the lungs is low, H^+^ will combine with HCO_3_^−^ to form carbonic acid, and then decompose into CO_2_ and water:

$$ {\mathrm{H}}^{+}+{{\mathrm{H}\mathrm{CO}}_3}^{-}\mathbf{\to}{\mathrm{H}}_2{\mathrm{CO}}_3\mathbf{\to}{\mathrm{CO}}_2+{\mathrm{H}}_2\mathrm{O} $$

After CO_2_ enters the lungs, it is eliminated by dissociation, which is the main way to reduce H^+^ concentration when CO_2_ is eliminated [1].

The amount and rate of gas exchange across the respiratory membrane are mainly determined by the partial pressure of each gas. The gas diffuses along the pressure gradient, from the part with the higher pressure to the lower pressure part. At standard atmospheric pressure, the partial pressure of oxygen (PO_2_) outside the body is greater than that inside the body after alveolar gas exchange. When the exercising muscles require more oxygen to meet metabolic needs, the venous oxygen is depleted and accelerates the alveolar gas exchange, resulting in PO_2_ reduction [1]. Therefore, O_2_ enters the blood and CO_2_ leaves the blood. PCO_2_ is mainly used to determine whether there is respiratory acidosis or alkalosis. Increased PCO_2_ suggests that there is insufficient lung ventilation, and CO_2_ retention in the body, which leads to respiratory acidosis. Lower PCO_2_, indicating hyperventilation (such as deeper or faster breathing), and excessive CO_2_ elimination in the body, leads to respiratory alkalosis [1]. Therefore, an increase in PCO_2_ will cause an increase in CO_2_ in the blood, which will result in a decrease in the pH value.

To sum up, the change of O_2_ and CO_2_ during long-term moderate-intensity exercise can reflect aerobic capacity to a certain extent. For that reason, the four variables (i.e., VO_2_, VCO_2_, PO_2_ and PCO_2_) were chosen to assess the influence of NaHCO_3_ on the oxidative system.

#### Overall meta-analysis of the oxidative system

**Figure 7:**
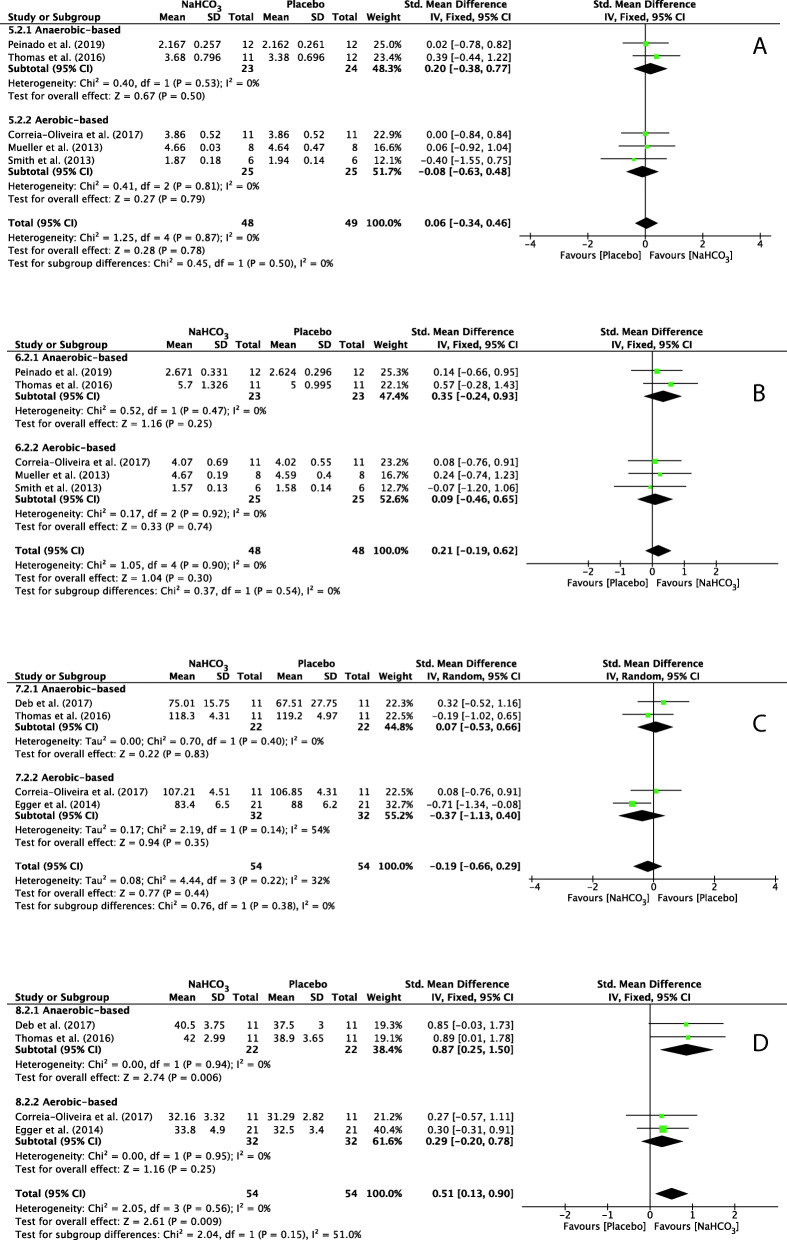
Fig. 7 Forest plot of standardized mean difference (SMD) of NaHCO_3_ vs. placebo on VO_2_(A), VCO_2_(B), PO_2_(C) and PCO_2_(D) after exercise. Squares represent the SMD for each study. The diamonds represent the pooled SMD for all studies. CI: Confidence interval, df: degrees of freedom

The forest plots depicting the individual SMDs and associated 95% CI and random-effect models for VO_2_, VCO_2_, PO_2_ and PCO_2_ are presented in Fig. 7.

The SMD for VO_2_ was 0.06 (95% CI: − 0.34 to 0.46), indicating a non-significant effect during exercise between NaHCO_3_ and placebo cohorts (*p* = 0.78) (Fig. 7a). Similarly, there was a non-significant effect during exercise after ingestion of NaHCO_3_ on VCO_2_ (SMD = 0.21, 95% CI: − 0.19 to 0.62, *P* = 0.30) and PO_2_ (SMD = − 0.19, 95% CI: − 0.66 to 0.29, *P* = 0.44) (Fig. 7b and c), but a significant effect on PCO_2_ (SMD = 0.51, 95% CI: 0.13 to 0.90, *P* = 0.009) (Fig. 7d). Heterogeneity was not detected among studies assessing VO_2_, VCO_2_ and PCO_2_ (I^2^ = 0%) and PO_2_ presented a low heterogeneity (I^2^ = 32%), shown in Fig. 7a, b, c and d respectively.

#### Sub-group analysis of the oxidative system

A sub-group analysis was performed to evaluate the effect of NaHCO_3_ ingestion on exercise with different metabolic characteristics. There was a non-significant difference between two cohorts for VO_2_ in anaerobic-based (SMD = 0.20, 95% CI: − 0.38 to 0.77, *P* = 0.50, I^2^ = 0%) and aerobic-based (SMD = − 0.08, 95% CI: − 0.63 to 0.48, *P* = 0.79, I^2^ = 0%) exercise (Fig. 7a). Similar to VCO_2_ and PO_2_, there was a non-significant difference between cohorts for VCO_2_ and PO_2_ in anaerobic-based exercise (SMD = 0.35, 95% CI: − 0.24 to 0.93, *P* = 0.25, I^2^ = 0% and SMD = 0.07, 95% CI: − 0.53 to 0.66, *P* = 0.83, I^2^ = 0% respectively) and aerobic-based exercise (SMD = 0.09, 95% CI: − 0.46 to 0.65, *P* = 0.74, I^2^ = 0% and SMD = − 0.37, 95% CI: − 1.13 to 0.40, *P* = 0.35, I^2^ = 54% respectively) (b and c in Fig. 7).

The opposite results are shown in Fig. 7d. There was a significant difference between cohorts for PCO_2_ in anaerobic-based (SMD = 0.87, 95% CI: 0.25 to 1.50, *P* = 0.006) but not aerobic-based (SMD = 0.29, 95% CI: − 0.20 to 0.78, P = 0.25) exercise. Heterogeneity was not detected among studies assessing PCO_2_ in anaerobic-based (I^2^ = 0%) and aerobic-based (I^2^ = 0%) exercise.

#### Strategic analysis of NaHCO3 on the oxidative system

**Figure 8:**
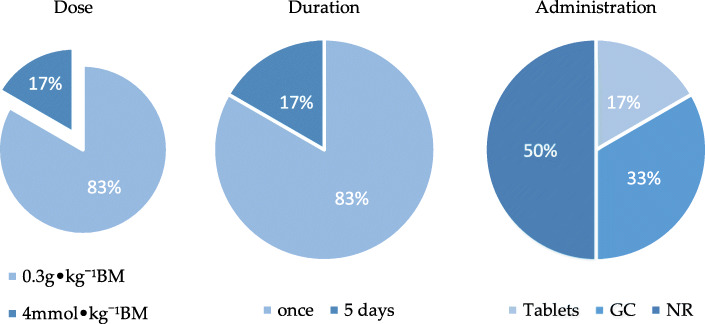
Fig. 8 The percentage of dose, duration and administration of 6 studies based on aerobic exercise. GC: gelatin capsules, NR: not record

For aerobic-based exercise (Table 7), 5 (83%) out of 6 studies used 0.3 g•kg^− 1^ BM of NaHCO3 and 1 article used 4 mmol•kg^− 1^ (about 0.336 g•kg^− 1^). The duration was once in 5 (83%) out of 6 studies, while 1 study had a duration of 5 consecutive days. The administration of NaHCO_3_ was in tablets in 1 study, gelatin capsules in 2 studies and not recorded in 3 studies (Fig. 8). Half of the studies showed NaHCO3 ingestion 90 min before the trial, other studies showed it 3–1.5 h before the trial.

## Discussion

To our knowledge, the present study is the first to assess the contribution of NaHCO_3_ ingestion on energy metabolism during exercise with a meta-analytic statistical technique using Review Manager 5.3 (v5.3, Cochrane Collaboration, Copenhagen, Denmark, 2020). The main findings of this analysis indicated that ingestion of NaHCO_3_ improves pH, HCO_3_^−^ and BE in the blood during exercise compared to a placebo (Figs. 2, 3, 4). However, BLa can be improved in anaerobic-based but not in aerobic-based exercise through ingestion of NaHCO_3_ (Fig. 5). Furthermore, compared to a placebo, ingestion of NaHCO_3_ during exercise does not improve VO_2_, VCO_2_ and PO_2_, although it improves PCO_2_ in anaerobic-based but not aerobic-based exercise (Fig. 7). Collectively these results indicate that ingestion of NaHCO_3_ is better than a placebo to improve AnMS but makes no difference to the oxidative system.

The discrepancies in the studies reported in this meta-analysis need to be considered. The extracellular to intracellular pH gradient increases as HCO_3_^−^ is impermeable to cellular membranes [50], resulting in a greater efflux of H^+^ and lactate from active muscles [51]. This occurs via either simple diffusion or by the lactate or H^+^ co-transporters [5]. It has been suggested that lactate efflux from muscles is higher as a result of extracellular alkalosis. However, Fig. 5 shows that there was no significant difference for BLa in an aerobic-based situation. That may explain the lack of effect with ingestion of NaHCO_3_ on performance that is based on the oxidative system, despite the significant effects on AnMS.

Therefore, a sensitivity analysis was performed to verify the results. According to the evaluation results in Table 5, the study with the lowest score [43] and another 5 articles [33, 41, 42, 44, 45] that were not given full marks were excluded. These sensitivity analysis results were similar to those of the original meta-analysis.

### Discussion on AnMS

Results in the present analysis indicate that NaHCO_3_ ingestion is effective in improving AnMS, which may be able to improve sport performance based on anaerobic capacity. The performance results of included studies showed that performance improved or was maintained the same when ingesting NaHCO_3_, while a placebo showed a decline in sport performance. (Table 6). This result is different from that of other meta-analyses [52, 53], but similar to several individual studies which did not meet the present eligibility criteria [54, 55]. Two included studies [32, 33] reported no improvement in sport performance, and we found that the experimental exercise in these two articles were more likely based on the ATP-CP system to obtain energy (Table 6). This is similar to previous studies [12, 56], where the ATP-CP system was not affected by NaHCO_3_ ingestion.

The key point of the contraindications in different results may be the gastrointestinal (GI) problems caused by ingestion of NaHCO_3_. Because the bicarbonate buffer system is not solely responsible for blood pH and is also vital in other systems, such as the stomach and duodenum by neutralizing gastric acid, abdominal pain and diarrhea are often experienced by individuals who take NaHCO_3_ [36, 57]. An article included in the present study also illustrated this problem [41]. While the results among all subjects indicated that the intake of NaHCO_3_ has no effect on sports performance, after excluding subjects who had GI problems with ingestion of NaHCO_3_, a significant difference in sports performance was observed. However, in this meta-analysis, the author extracted the data of all subjects from this article and verified that it did not affect the results of the meta-analysis. In response to the GI problems, some countermeasures have been taken that have been scientifically proven to alleviate or prevent GI problems. For example, ingestion of a large amount of water [58], with food [9], with carbohydrate [59] or administration as enteric-formulated capsules [60]. More measures to prevent GI problems may help demonstrate the improvement in sport performance with the intake of NaHCO_3_ as subjects are not troubled by GI problems.

#### Discussion on the oxidative system

Although the overall PCO_2_ in Fig. 7 shows a significant difference, aerobic-based exercise alone presented no significant difference. Therefore, ingestion of NaHCO_3_ does not benefit exercise based on the oxidative system, which means it may not be able to improve sport performance that is based on aerobic capacity. This is similar to the performance results shown in Table 7, with the exception of one study [45] that did not record performance results and two other studies that had results possibly due to chronic ingestion [46], or the decrease in PO_2_ [47] due to ingestion of NaHCO_3_. As mentioned before, PO_2_ reduction accelerates alveolar gas exchange. The results based on this meta-analysis, that the sport performance based on aerobic capacity is not affected by NaHCO_3_ ingestion, is different from some previous studies [14, 61], but similar to other studies [15, 62].

There is a reason why NaHCO_3_ intake will cause different results for aerobic-based exercise. Whether ATP is produced under aerobic or anaerobic conditions, glycogen plays an important role. Glycogen can provide energy to maintain moderate-intensity exercise for 3 to 5 min under aerobic conditions. The reason some studies [14, 61] have different results from the present study may be because they are based on the aerobic energy supply form of muscle glycogen. However, the studies included in this present meta-analysis are based on the aerobic energy supply form of fat (according to the exercise time, energy from fat can be maintained for 1–2 h or more) (Table 1). Different forms of the oxidative system supply may be one of the reasons for the different performance results after ingestion of NaHCO_3_.

#### Limitations

A number of limitations may be present in this meta-analysis and should be considered. Firstly, the choice of variables that reflect the ATP-CP, glycolytic and oxidative systems may not be a good representative of performance. As we know, the substrates of ATP recovery for the ATP-CP, glycolytic and oxidative system are ATP/PCr, glucose and fat (i.e. free fatty acid [FFA], which are the main energy sources for the oxidative system) [1] respectively. The ideal way is to use these variables because the changes in their content can directly reflect the changes in the capacity of each energy metabolism system. However, a total of 9 articles from the initial search analyzed using these parameters (i.e., ATP, PCr, glucose or FFA), and there was only one left after excluding articles that did not meet the eligibility criteria. This is why we chose pH, HCO_3_^−^, BE and BLa; VO_2_, VCO_2_, PO_2_ and PCO_2_ that reflect the changes in the capacity of each energy metabolism system indirectly instead, which may affect the accuracy of the research results.

Additionally, this study analyzes the integration of the ATP-CP and glycolytic system as an AnMS, but in fact the research results of this article may be biased towards the glycolytic system. ATP resynthesis into ATP-CP occurs very quickly, and intake of NaHCO_3_ may be too late to have an effect. Therefore, there is a lack of a specific influence of ingestion of NaHCO_3_ on the ATP-CP system, while other studies have reported that induced alkalosis does not affect the ATP-CP system, but does benefit the glycolytic system and does not impact the oxidative system [11, 17], similar to the results in the present meta-analysis.

## Conclusions

This meta-analysis provides evidence that ingestion of NaHCO_3_ increases the content of pH, HCO_3_^−^, BE and lactate in the blood, that may be beneficial to exercise based on the anaerobic metabolism system, especially based on the glycolytic system. The ideal way is to ingest it in a gelatin capsule in an acute mode and use a dose of 0.3 g•kg^− 1^ BM of NaHCO_3_ 90 min before the trial. Furthermore, the specific form of aerobic oxidative supply should be considered before ingesting NaHCO_3_ when doing aerobic exercise. Therefore, athletes and coaches should take notice that anaerobic and aerobic exercise and sports capacity based on the glycolytic system may be improved by supplementing with NaHCO_3._

## Data Availability

The data used and/ or analyzed during the current study are available from the corresponding author on reasonable request.

## Abbreviations

NaHCO_3_: Sodium bicarbonate
HCO_3_^−^: Bicarbonate ion
BE: Base excess
BLa: Blood lactate
VO_2_: Oxygen uptake
VCO_2_: Carbon dioxide production
PO_2_: Partial pressure of oxygen
PCO_2_: Partial pressure of carbon dioxide
AnMS: Anaerobic metabolism system
ATP: Adenosine triphosphate
CP: Creatine phosphate
PCr: Phosphocreatine
ADP: Adenosine diphosphate
CUE: Continuous unit of energy
SMD: Standardized mean differences
SE: Standard error
SD: Standard deviation
CI: Confidence interval
GI: Gastrointestinal
FFA: Free fatty acid

## Footnotes

1 https://apps.automeris.io/wpd/index.zh_CN.html
